# Risk factors and 180-day mortality of acute kidney disease in critically ill patients: A multi-institutional study

**DOI:** 10.3389/fmed.2023.1153670

**Published:** 2023-04-17

**Authors:** Heng-Chih Pan, Hsing-Yu Chen, Hui-Ming Chen, Yu-Tung Huang, Ji-Tseng Fang, Yung-Chang Chen

**Affiliations:** ^1^Chang Gung University College of Medicine, Taoyuan, Taiwan; ^2^Division of Nephrology, Department of Internal Medicine, Keelung Chang Gung Memorial Hospital, Keelung, Taiwan; ^3^Graduate Institute of Clinical Medical Sciences, College of Medicine, Chang Gung University, Taoyuan, Taiwan; ^4^Division of Chinese Internal Medicine, Center for Traditional Chinese Medicine, Chang Gung Memorial Hospital, Taoyuan, Taiwan; ^5^School of Traditional Chinese Medicine, College of Medicine, Chang Gung University, Taoyuan, Taiwan; ^6^Center for Big Data Analytics and Statistics, Chang Gung Memorial Hospital, Linkou Medical Center, Taoyuan, Taiwan; ^7^Division of Nephrology, Department of Internal Medicine, Linkou Chang Gung Memorial Hospital, Taoyuan, Taiwan

**Keywords:** acute kidney injury, acute kidney disease, chronic kidney disease, risk factor, survival

## Abstract

**Background:**

Critically ill patients with acute kidney injury (AKI) have a poor prognosis. Recently, the Acute Disease Quality Initiative (ADQI) proposed to define acute kidney disease (AKD) as acute or subacute damage and/or loss of kidney function post AKI. We aimed to identify the risk factors for the occurrence of AKD and to determine the predictive value of AKD for 180-day mortality in critically ill patients.

**Methods:**

We evaluated 11,045 AKI survivors and 5,178 AKD patients without AKI, who were admitted to the intensive care unit between 1 January 2001 and 31 May 2018, from the Chang Gung Research Database in Taiwan. The primary and secondary outcomes were the occurrence of AKD and 180-day mortality.

**Results:**

The incidence rate of AKD among AKI patients who did not receive dialysis or died within 90 days was 34.4% (3,797 of 11,045 patients). Multivariable logistic regression analysis indicated that AKI severity, underlying early CKD, chronic liver disease, malignancy, and use of emergency hemodialysis were independent risk factors of AKD, while male gender, higher lactate levels, use of ECMO, and admission to surgical ICU were negatively correlated with AKD. 180-day mortality was highest among AKD patients without AKI during hospitalization (4.4%, 227 of 5,178 patients), followed by AKI with AKD (2.3%, 88 of 3,797 patients) and AKI without AKD (1.6%, 115 of 7,133 patients). AKI with AKD had a borderline significantly increased risk of 180-day mortality (aOR 1.34, 95% CI 1.00–1.78; *p* = 0.047), while patients with AKD but no preceding AKI episodes had the highest risk (aOR 2.25, 95% CI 1.71–2.97; *p* < 0.001).

**Conclusion:**

The occurrence of AKD adds limited additional prognostic information for risk stratification of survivors among critically ill patients with AKI but could predict prognosis in survivors without prior AKI.

## 1. Introduction

Acute kidney injury has been recognized as a major global health concern (1, 2). Due to the aging population and increase in associated comorbidities, the incidence of acute kidney injury (AKI) is growing among ICU patients (3). The pathophysiology of AKI is multifactorial and is also attributed to dysfunction in other organs, such that AKI is often involved in multiple organ failure syndrome (4). In the literature, the occurrence of AKI is associated with higher risk of multiple comorbidities, chronic kidney disease (CKD), and short-and long-term mortality (4–8). However, kidney damage may persist after an episode of AKI has ended. Recently, the Acute Disease Quality Initiative (ADQI) proposed to define acute kidney disease (AKD) as acute or subacute damage and/or loss of kidney function that lasts 7–90 days post AKI (9).

AKD can include patients with evolving kidney disease that might not fulfil the strict criteria for AKI, and patients with AKI who have prolonged kidney dysfunction but do not fulfil the criteria for CKD (10). AKD is a growing concern; several previous studies had shown that AKD without AKI, and AKD following AKI, are both associated with significantly worse outcomes compared to AKI patients with renal recovery (1, 2, 11–15). Nevertheless, the epidemiology of critically ill patients with AKD after AKI is still very limited. Furthermore, whether the occurrence of AKD adds additional prognostic information after AKI remains unclear.

To address the knowledge gaps in the current understanding of AKD, we conducted a multi-institutional study of critically ill patients in Taiwan. The purpose of this study was to identify critically ill patients who are likely to develop AKD, and to further examine whether the occurrence of AKD adds prognostic information in addition to AKI stage in predicting 180-day mortality.

## 2. Materials and methods

### 2.1. Data source

We used the Chang Gung Research Database (CGRD) as the data source in this study. The CGRD is a collection of daily medical records that have been prospectively collected from seven branches of Chang Gung Memorial Hospital in Taiwan since January 2001 and covers an annual average of 500,000 emergency department visits, 8,500,000 outpatient visits, and over 280,000 admissions to 10,070 beds. In 2015, outpatient and inpatient records comprised 6.1 and 10.2% of the CGRD, respectively (16). The abundance of medical information available from the CGRD makes it a good source for the conduct of retrospective clinical studies (16–18). The CGRD collects the patient’s personal information, including gender, body weight, height, lifestyle, and birth date. In addition, laboratory findings (e.g., serum creatinine [sCr], blood nitrogen urea [BUN], etc.), results of imaging exams, and comprehensive information regarding every emergency visit, inpatient admission, and outpatient visit, are available in the CGRD. The International Classification of Diseases, 9th and 10th revision, Clinical Modification (ICD-9-CM and ICD-10-CM) codes were used to categorize patients’ underlying diseases and the reasons for admission and emergency and outpatient visits. This information was stored digitally. The chart number of each patient was securely encrypted to protect personal privacy and was only used to link data between different databases in the CGRD.

### 2.2. Study design

**Figure 1:**
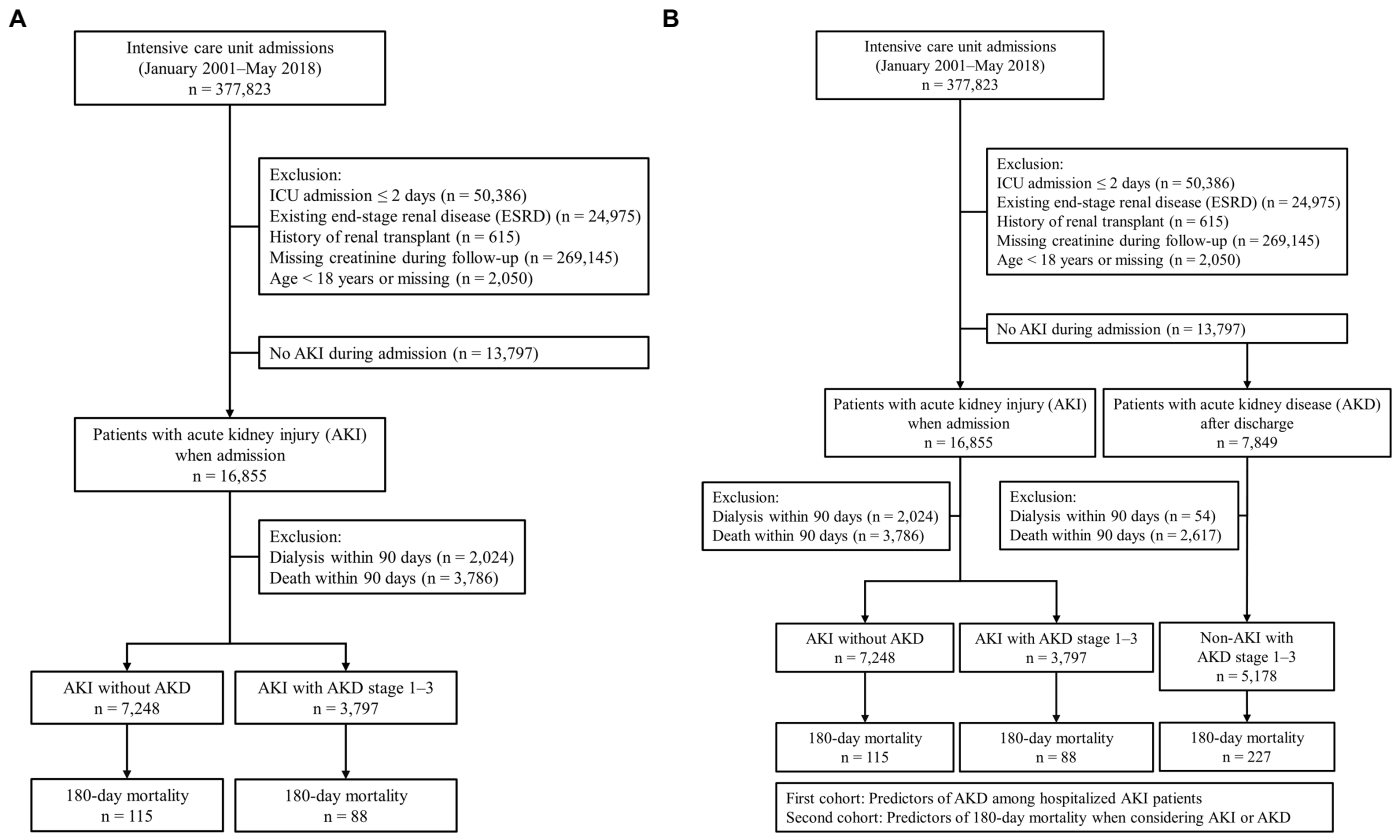
Flowchart showing the study protocol. **(A)** First cohort and **(B)** second cohort.

The study protocol is shown in Figure 1, and the data were retrospectively analyzed. All admissions to the intensive care unit (ICU) between January 2001 and May 2018 were obtained from the CGRD (*n* = 377,823). Subjects excluded from analysis were those with ICU admission for less than 2 days (*n* = 50,386), existing ESRD (*n* = 24,975), history of kidney transplant (*n* = 615), age outside the limits set in this study and/or missing age data (*n* = 2,050), and missing sCr data during the 180-day follow-up period (*n* = 269,145). Two cohorts were defined in this study. The first cohort consisted of ICU patients who had survived AKI (*n* = 11,045) and was used to explore the predictive covariates for the occurrence of AKD. The second cohort consisted of AKD patients without prior AKI during ICU admission. Patients from both cohorts were used to explore predictors of 180-day mortality in the following clinical scenarios: AKI without AKD, AKI with AKD stage 1–3, and AKD stage 1–3 with no AKI. AKI was diagnosed and classified according to the Kidney Disease: Improving Global Outcomes (KDIGO) definition, and stages were defined by the ratio of the lowest sCr value during the first 7 days of ICU admission to the highest sCr value within 3 months prior to ICU admission. Based on the results of our previous study, ratios of 1.5–2, 2–3, and >3 were used to classify AKI stage 1, 2, and 3, respectively (4). Patients who required urgent hemodialysis were also defined as having stage 3 AKI (19). The definition of AKD in this study was based on the consensus of the ADQI 16 workgroup in 2017. Fold increases of <1.5, 1.5–2, 2–3, and >3 in sCr level from baseline after AKI injury indicated AKD stage 0, 1, 2, and 3, respectively (10). Since sCr levels may fluctuate over a period of 7–90 days post AKI injury, the lowest sCr value during this period was used for the diagnosis of AKD. The 180-day mortality was defined as death of the patient, or discharge against medical advice due to being in a critically ill condition, at 90–180 days after diagnosis of AKI. To avoid potential confounding bias, patients undergoing dialysis and those who died within 90 days after AKI diagnosis were excluded. The entire study protocol was approved by the Institutional Review Board of the Chang Gung Medical Foundation (IRB No.: 201702274B0).

### 2.3. Covariates

Diabetes mellitus (DM; 250, E10, E11), hypertension (401, 402, E10, E11), cardiovascular diseases (410, 427.31, 428, 430, 431, 433, 434, I21, I48.0, I48.2, I48.91, I50, I60, I61, I65 and I66), chronic liver diseases (571, K70), malignancies (140–175, 179–208, C00-C97 and D00-D48), and CKD (580–589, I12, I13, N00-N05, N07, N11, N14, N17,-N19 and Q61) were recognized as comorbidities if patients had at least two outpatient visits or one inpatient admission with these diagnosis codes within one year preceding the date of ICU admission (Supplementary Table S1). To assess the possible seasonal variations, the four seasons in Taiwan were divided into “spring to summer” (March to August) and “autumn to winter” (September to February). Laboratory data obtained within the first 7 days of ICU admission included sCr, serum sodium, serum albumin, fasting sugar, hemoglobin, platelet count, leukocytes, procalcitonin, c-reactive protein (CRP), lactate, B-type natriuretic peptide (BNP), total bilirubin, and prothrombin time. If multiple test results were available, the first result was used. The estimated glomerular filtration rate (eGFR) was calculated from the sCr value according to the Modification of Diet in Renal Disease equation, and the eGFR value was used to classify the CKD stages. Medication records were also collected, including angiotensin-converting enzyme inhibitors (ACEi), angiotensin receptor blockers (ARB), direct renin inhibitors (DRI), and calcium channel blockers (CCB). Patients who used these medications within one year preceding the ICU admission date were recognized as users. In addition, the qSOFA score was assessed to classify the initial disease severity at ICU admission (20). Shock and anemia were defined as mean arterial pressure ≤ 65 mmHg and hemoglobin ≤10 g/dl within 7 days before ICU admission, separately. The proportion of use of radiocontrast within 7 days before ICU admission was also obtained as the possible existence of radiocontrast-induced nephropathy. Additionally, ICU procedures that could influence renal function, including emergency hemodialysis, extracorporeal membrane oxygenation (ECMO), ventilator use, coronary artery bypass grafting (CABG), intra-aortic balloon pump (IABP), and other emergency surgeries, were recorded, as was the use of norepinephrine and dopamine during ICU admission.

### 2.4. Statistical analysis

Descriptive statistics were used to present the baseline differences between AKI patients with and without AKD. Categorical covariates were presented as numbers and proportions and were analyzed with *χ*^2^ tests. Continuous covariates were presented as medians with interquartile ranges (IQRs), and Mann–Whitney U tests were used to examine the differences between AKD and non-AKD patients. Univariable and multivariable logistic regression was used to evaluate the predictors for AKD occurrence among AKI patients (cohort 1), and to evaluate the odds ratios of 180-day mortality among the three different conditions in cohort 2, including AKD stage 0–3, AKI stage 0–3, and AKI without AKD, AKI with AKD, and AKD without AKI. The validity of the regression model predicting AKD, and 180-day mortality was examined by C-statistics. All statistical analyses were carried out using SAS v 9.1.3 (SAS Institute, Cary, NC) and results with value of *p* <0.05 were regarded as statistically significant.

## 3. Results

### 3.1. Demographic features

Between 1 January 2001 and 31 May 2018, a total of 377,823 subjects were admitted to ICU, and 16,855 AKI events (55.1%) were identified among all eligible subjects. Within 90 days after ICU admission, 2,024 subjects received permanent dialysis and 3,786 subjects had died. A total of 11,045 AKI subjects entered the final analysis stage; stage 1 AKI was most prevalent (*n* = 7,284, 65.9%), followed by stage 3 (*n* = 2,152, 19.5%) and stage 2 AKI (*n* = 1,609, 14.6%). CKD was the most common comorbidity observed among all AKI patients (*n* = 6,081, 55.1%), and most had stage 3–5 CKD (*n* = 3,562, 32.2%). Most patients had hypertension (*n* = 5,617, 50.9%) and cardiovascular diseases (*n* = 4,400, 39.9%). During hospitalization, more than 90% of AKI patients were admitted to the non-surgical ICU (10,333, 93.6%), and more than 80% of patients were at low risk of multiple organ failure (qSOFA score 0–1 upon ICU admission, *n* = 8,993, 81.4%). However, nearly half of the patients needed ventilator support (*n* = 4,941, 44.7%). On the other hand, there were no differences in admission season, shock, anemia, or use of radiocontrast before admission.

### 3.2. Covariates associated with the occurrence of AKD

After discharge, 7,248 AKI patients (65.6%) had completely recovered, whereas 3,797 patients were diagnosed with AKD stage 1–3 (34.4%, Table 1). AKD patients had a higher proportion of females than non-AKD patients (37.5% versus 31.9%, *p* < 0.001). AKD patients tended to be at higher AKI stages than non-AKD patients (55, 20, 25% versus 71.7, 11.7, and 16.6% for AKI stage 1, 2 and 3, respectively; *p* < 0.001). For comorbidities, a lower proportion of AKD patients had hypertension (46.6%) and cardiovascular diseases (37.2%) compared to non-AKD patients (53.1 and 41.2%, respectively, *p* < 0.001). However, compared to patients without AKD, there were more AKD patients with pre-existing stage 3–5 CKD (31.2% versus 34.2%, *p* < 0.001). AKD patients had marginally lower serum creatinine (1.47, IQR: 1.70 mg/dl) than non-AKD patients (1.52, IQR: 1.44 mg/dl, *p* < 0.001), and lower lactate levels (20, IQR: 25.9 mg/dl versus 23.7, IQR: 32.9 mg/dl; *p* < 0.001). No significant differences in medications were found between AKD and non-AKD patients. A greater proportion of AKD patients were admitted to the non-surgical ICU compared to non-AKD patients (95.3% versus 92.6%, *p* < 0.001), and more AKD patients were classified as low risk upon ICU admission (83.8% versus 80.2%, *p* < 0.001) (Table 2). Additionally, fewer AKD patients needed ventilator support (40.9% versus 46.7%, *p* < 0.001) or ECMO (4.0% versus 6.0%, *p* < 0.001). However, emergency hemodialysis was more common among AKD patients (4.8% versus 3.1%, *p* < 0.001), as was dopamine use (28.2% in AKD patients and 22.9% in non-AKD patients; *p* < 0.001).

**Table 1:** Demographic characteristics of AKI patients with or without AKD upon ICU admission.

Covariates	No AKD (*n* = 7,248)		AKD stage 1–3 (*n* = 3,797)		*p* value
Gender					<0.001
Female (*n*, %)	2,314	31.9	1,423	37.5	
Male (*n*, %)	4,934	68.1	2,374	62.5	
Age, years (mean, SD)	64.4	15.3	64.6	15.4	0.431
ICU admission season					0.148
Spring and summer (March to August)	3,646	50.3	1,965	51.8	
Autumn and winter (September to February)	3,602	49.7	1,832	48.2	
AKI stage at ICU admission					< 0.001
Stage 1 (*n*, %)	5,197	71.7	2,087	55.0	
Stage 2 (*n*, %)	848	11.7	761	20.0	
Stage 3 (*n*, %)	1,203	16.6	949	25.0	
Comorbidities					
DM (*n*, %)	2,745	37.9	1,436	37.8	0.956
Hypertension (*n*, %)	3,846	53.1	1,771	46.6	< 0.001
Cardiovascular diseases (*n*, %)	2,988	41.2	1,412	37.2	< 0.001
Chronic liver diseases (*n*, %)	885	12.2	729	19.2	< 0.001
Malignancies (*n*, %)	783	10.8	605	15.9	< 0.001
CKD					< 0.001
No CKD (*n*, %)	3,198	44.1	1,766	46.5	
Stage 1–2 (*n*, %)	1,788	24.7	731	19.3	
Stage 3–5 (*n*, %)	2,262	31.2	1,300	34.2	
Biochemical profiles					
Creatinine, mg/dL (median, IQR)	1.52	1.44	1.47	1.7	< 0.001
Sodium, mmol/L (median, IQR)	139	7	138	6	< 0.001
Albumin, g/dL (median, IQR)	2.8	0.9	2.7	0.8	< 0.001
Fasting sugar, mg/dL (median, IQR)	175	111	166	110	0.007
Hemoglobin, g/dL (median, IQR)	10.2	2.5	9.9	2.6	< 0.001
Platelet, 10^9^/L (median, IQR)	111	75	104	80	0.005
Leukocytes, 10^9^/L (median, IQR)	6.9	8.3	6.7	8.3	0.445
Procalcitonin, ng/mL (median, IQR)	2.86	12.82	2.08	11.13	0.044
CRP, mg/L (median, IQR)	75.20	119.7	63.73	114.68	< 0.001
Lactate, mg/dL (median, IQR)	23.7	32.9	20	25.9	< 0.001
BNP, pg./mL (median, IQR)	770.0	1,159	836.6	1,482	0.009
Total bilirubin, mg/dL (median, IQR)	1.00	1.1	1.00	1.4	0.016
Prothrombin time, sec (median, IQR)	11.9	2.8	12.3	3.4	<0.001
Medication					
ACEi (*n*, %)	987	13.6	480	12.6	0.151
ARB (*n*, %)	1,440	19.9	721	19.0	0.269
DRI (*n*, %)	38	0.5	12	0.3	0.122
CCB (*n*, %)	1,966	27.1	1,020	26.9	0.769
180-day mortality	115	1.59	88	2.32	0.007

ACEi, angiotensin converting enzyme inhibitor; ARB, angiotensin II receptor blocker; AKD, acute kidney disease; AKI, acute kidney injury; BNP, B-type natriuretic peptide; CCB, calcium channel blocker; CKD, chronic kidney disease; CRP, C-reactive protein; DM, diabetes mellitus; DRI, direct renin inhibitor; SD, standard deviation.

**Table 2:** Association between disease severity upon ICU admission and AKD stages.

Covariates	No AKD (*n* = 7,248)		AKD stage 1–3 (*n* = 3,797)		*p* value
Intensive care units					
Surgical ICU (*n*, %)	534	7.4	178	4.7	<0.001
Non-surgical ICU (*n*, %)	6,714	92.6	3,619	95.3	
Illness at ICU admission					
Shock (*n*, %)	525	7.2	289	7.6	0.482
Anemia (*n*, %)	1,428	19.7	776	20.4	0.359
Use of radiocontrast before ICU admission (*n*, %)	428	5.9	234	6.2	0.588
qSOFA					<0.001
Low risk (0–1) (*n*, %)	5,813	80.2	3,180	83.8	
High risk (2–3) (*n*, %)	1,435	19.8	617	16.3	
Hospitalization, days (mean, SD)	27.0	15.9	31.1	17.3	<0.001
Procedures					
Emergency hemodialysis (*n*, %)	225	3.1	181	4.8	<0.001
ECMO (*n*, %)	432	6.0	153	4.0	<0.001
Ventilator use (*n*, %)	3,387	46.7	1,554	40.9	<0.001
CABG (*n*, %)	653	9.0	224	5.9	<0.001
IABP (*n*, %)	294	4.1	154	4.1	0.999
Emergency surgery (*n*, %)	3,585	49.5	1,657	43.6	<0.001
Medications					
Norepinephrine (*n*, %)	981	13.5	530	14.0	0.538
Dopamine (*n*, %)	1,662	22.9	1,071	28.2	<0.001

AKD, acute kidney disease; CABG, coronary artery bypass graft; CCU, coronary care unit; ECMO, extracorporeal membrane oxygenation; IABP, intra-aortic balloon pump; MICU, medical intensive care unit; qSOFA, Quick Sequential Organ Failure Assessment; SD, standard deviation; SICU, surgical intensive care unit.

Table 3 shows the unadjusted and adjusted odds ratio (OR, aOR) for AKD occurrence. When considering all covariates, a higher AKI stage was associated with the occurrence of AKD (aOR 2.15, 95% CI 1.92–2.41 for stage 2 AKI, *p* < 0.001; aOR 1.91, 95% CI 1.72–2.12 for stage 3 AKI, *p* < 0.001). Male gender was associated with a 22% lower risk of AKD occurrence (95% CI 0.72–0.85), and the older patients (aged ≥70 years) had a 1.16-fold higher risk (95% CI 1.06–1.27) than younger patients (aged <70 years). In addition, patients with early CKD had a 1.46-fold higher risk of AKD than patients with no pre-existing CKD (95% CI 1.23–1.73; *p* < 0.001). On the other hand, hypertension (aOR 0.83, 95% CI 0.76–0.91; *p* < 0.001) and cardiovascular diseases (aOR 0.91, 95% CI 0.83–0.99; *p* = 0.047) lowered the risk of AKD by 17 and 9%, respectively. Pre-existing chronic liver diseases (aOR 1.43, 95% CI 1.27–1.61; *p* < 0.001) and malignancies (aOR 1.30, 95% CI 1.14–1.47; *p* < 0.001) increased the risk of AKD occurrence by 43 and 30%, respectively. During ICU hospitalization, higher qSOFA scores (aOR 0.87, 95% CI 0.77–0.97; *p* = 0.017) and higher lactate levels (aOR 0.74, 95% CI 0.63–0.86; *p* < 0.001) were associated with lower risk of AKD. In addition, emergency hemodialysis (aOR 1.29, 95% CI 1.04–1.59; *p* = 0.019), and use of dopamine (aOR 1.14, 95% CI 1.03–1.25; *p* = 0.009) raised the risk of AKD by 29 and 14%, respectively, whereas ECMO (aOR 0.76, 95% CI 0.62–0.94; *p* = 0.012) and ventilator support (aOR 0.84, 95% CI 0.77–0.93; *p* < 0.001) decreased the risk of AKD.

**Table 3:** Univariable and multivariate logistic regression of factors associated with the occurrence of AKD.

Covariates	Univariable			Multivariable		
	OR	95% CI	*p* value	aOR	95% CI	*p* value
AKI stage at ICU admission						
Stage 1	ref			ref		
Stage 2	2.24	(2.00–2.50)	<0.001	2.15	(1.92–2.41)	<0.001
Stage 3	1.96	(1.78–2.17)	<0.001	1.91	(1.72–2.12)	<0.001
Gender						
Female	ref			ref		
Male	0.78	(0.72–0.85)	<0.001	0.78	(0.72–0.85)	<0.001
Age group						
<70 years	ref			ref		
≥ 70 years	1.06	(0.98–1.15)	0.155	1.16	(1.06–1.27)	<0.001
ICU admission season						
Spring and summer (March to August)	ref			ref		
Autumn and winter (September to February)	0.94	(0.87–1.02)	0.148	0.95	(0.88–1.03)	0.236
Hospitalization						
<25 days	ref			ref		
≥ 25 days	1.54	(1.42–1.67)	<0.001	1.48	(1.36–1.61)	<0.001
Cause						
Non-surgical ICU	ref					
Surgical ICU	0.62	(0.52–0.74)	<0.001	0.65	(0.54–0.78)	<0.001
Comorbidities						
DM	1.00	(0.92–1.08)	0.956	1.00	(0.92–1.10)	0.989
Hypertension	0.77	(0.72–0.84)	<0.001	0.83	(0.76–0.91)	<0.001
Cardiovascular diseases	0.84	(0.78–0.92)	<0.001	0.91	(0.83–0.99)	0.047
Chronic liver diseases	1.71	(1.54–1.90)	<0.001	1.43	(1.27–1.61)	<0.001
Malignancies	1.57	(1.40–1.75)	<0.001	1.30	(1.14–1.47)	<0.001
CKD						
No CKD	ref			ref		
Stage 1–2	1.50	(1.27–1.76)	<0.001	1.46	(1.23–1.73)	<0.001
Stage 3–5	1.15	(1.01–1.31)	0.042	1.01	(0.87–1.16)	0.919
qSOFA						
Low risk (0–1)	ref			ref		
High risk (2–3)	0.79	(0.71–0.87)	<0.001	0.87	(0.77–0.97)	0.017
Biochemical profiles						
Albumin (<2.8 g/dl vs. ≥ 2.8 g/dl)	1.44	(1.33–1.57)	<0.001	1.18	(1.08–1.29)	<0.001
Total bilirubin (> 1.4 mg/dl vs. ≤ 1.4 mg/dl)	1.28	(1.14–1.44)	<0.001	0.97	(0.85–1.10)	0.612
Lactate (> 19.8 mg/dl vs. ≤ 19.8 mg/dl)	0.75	(0.65–0.87)	<0.001	0.74	(0.63–0.86)	<0.001
Prothrombin (> 12 s vs. ≤ 12 s)	1.83	(1.40–2.40)	<0.001	1.50	(1.12–1.99)	0.006
Illness at ICU admission						
shock (*n*, %)	1.06	(0.91–1.23)	0.479	0.98	(0.84–1.15)	0.845
anemia (*n*, %)	1.05	(0.95–1.16)	0.357	0.98	(0.89–1.09)	0.725
Use of radiocontrast before ICU admission (*n*, %)	1.05	(0.89–1.23)	0.586	1.04	(0.87–1.23)	0.667
Procedures during ICU admission						
Emergency hemodialysis	1.56	(1.28–1.91)	<0.001	1.29	(1.04–1.59)	0.019
ECMO	0.66	(0.55–0.80)	<0.001	0.76	(0.62–0.94)	0.012
Use of ventilator	0.79	(0.73–0.86)	<0.001	0.84	(0.77–0.93)	<0.001
CABG	0.63	(0.53–0.74)	<0.001	0.86	(0.71–1.04)	0.124
IABP	1.00	(0.82–1.22)	1.000	1.36	(1.08–1.71)	0.008
Emergency surgery	0.79	(0.73–0.86)	<0.001	0.87	(0.79–0.95)	0.003
Medication						
Norepinephrine	1.04	(0.93–1.16)	0.537	0.92	(0.81–1.05)	0.218
Dopamine	1.32	(1.21–1.44)	<0.001	1.14	(1.03–1.25)	0.009

AKD, acute kidney disease; AKI, acute kidney injury; CABG, coronary artery bypass graft; CKD, chronic kidney disease; DM, diabetes mellitus; ECMO, extracorporeal membrane oxygenation; IABP, intra-aortic balloon pump; ICU, intensive care unit; qSOFA, Quick Sequential Organ Failure Assessment.

### 3.3. Association between different AKD conditions and 180-day mortality

**Figure 2:**
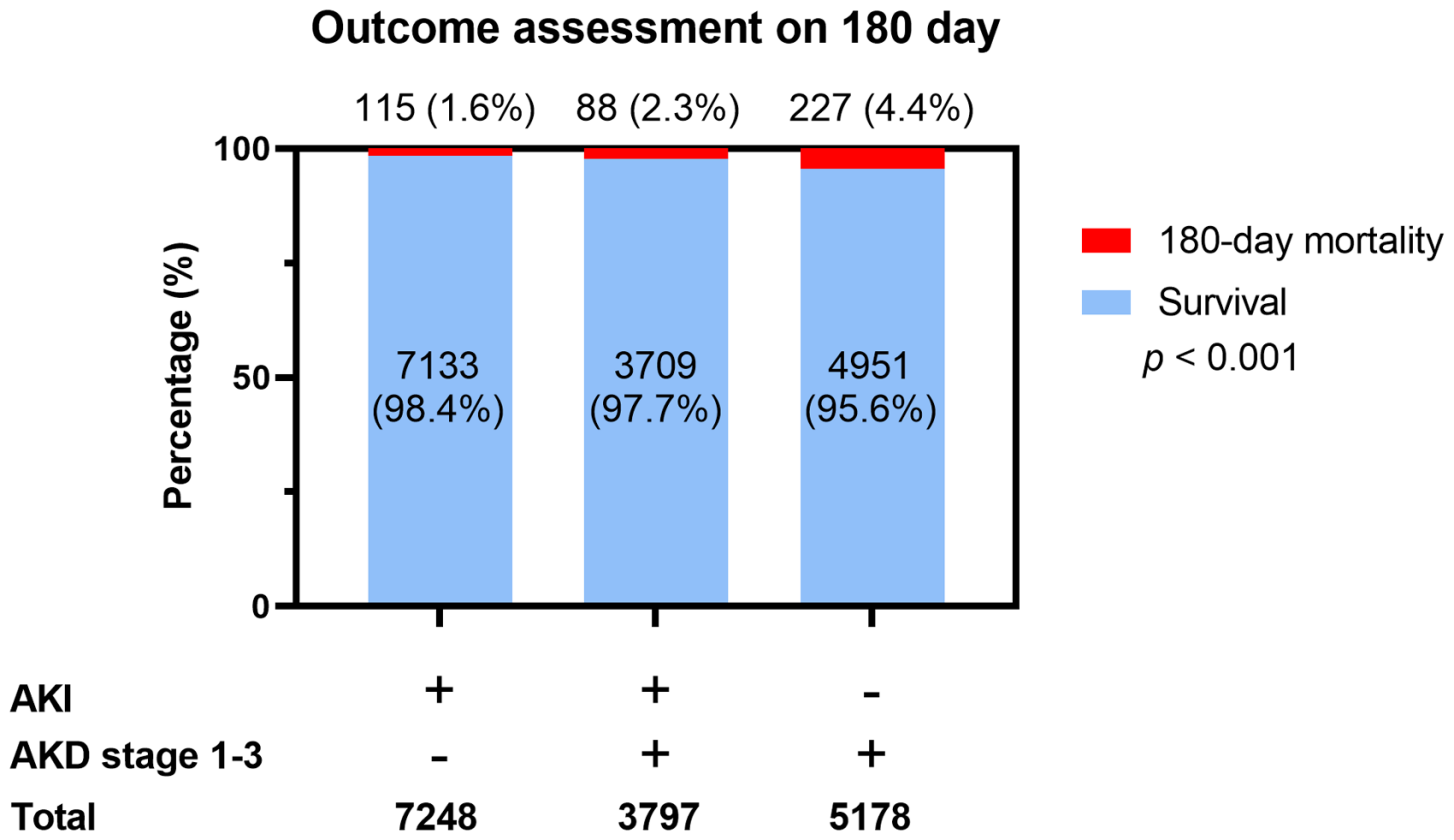
Assessment outcome among patients with different combinations of AKI and AKD.

**Figure 3:**
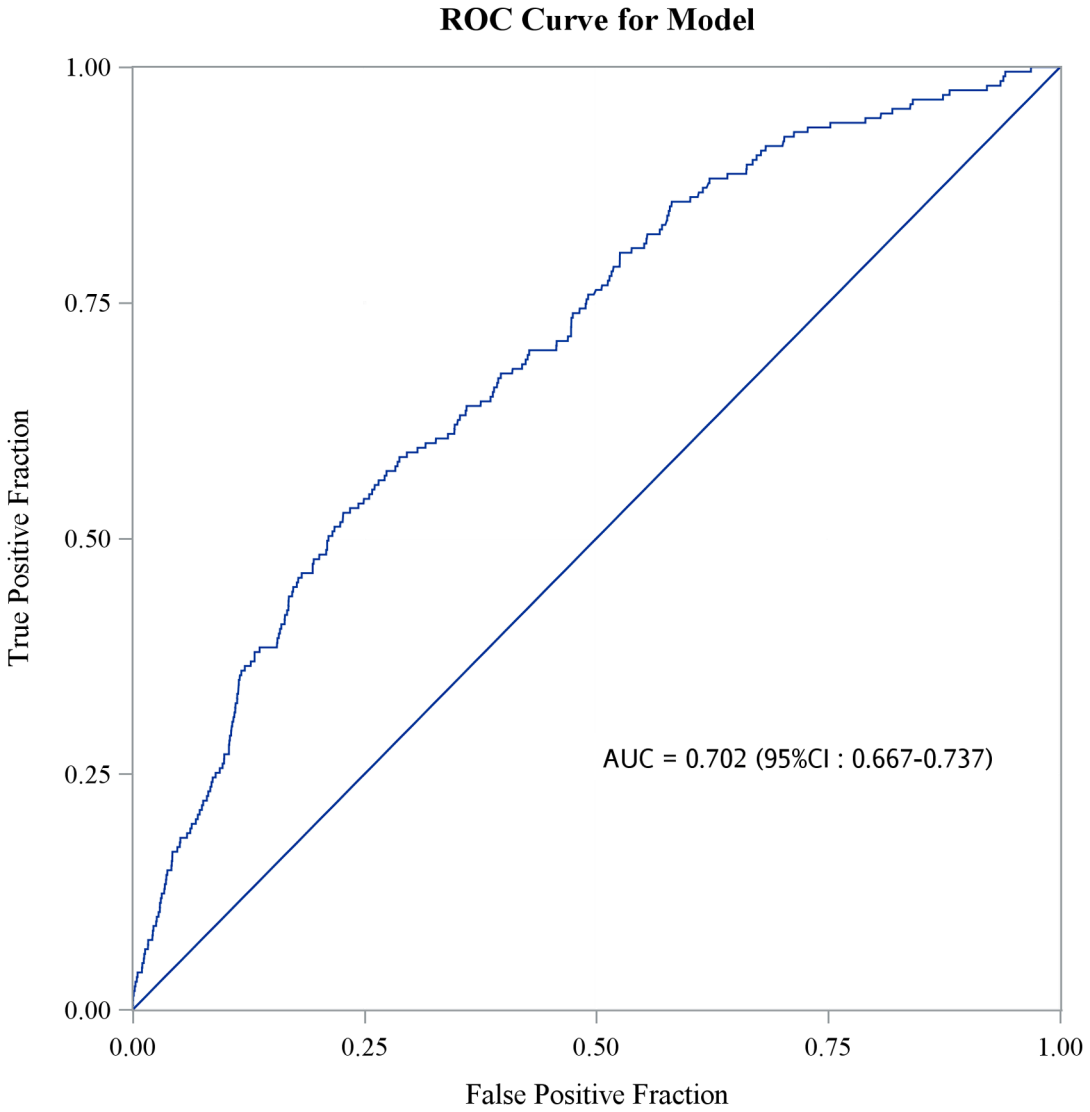
Discrimination ability of models predicting the occurrence of 180-day mortality.

Figure 2 shows the 180-day outcomes associated with the various AKI/AKD conditions. In addition to 11,045 hospitalized AKI patients, we enrolled 5,178 patients without prior AKI events who experienced AKD within 90 days of ICU admission. The 180-day mortality rate appeared to be highest among patients with AKD without AKI events (*n* = 227, 4.4% of 5,178 patients), followed by AKD patients with AKI (*n* = 88, 2.3% of 3,797 patients) and AKI patients without AKD (*n* = 115, 1.6% of 7,248 patients, *p* < 0.001). A higher proportion of patients who died after 180 days consisted of those whose hospital stays exceeded 25 days and those with chronic liver diseases and malignancies, but fewer patients in this group had cardiovascular diseases (Supplementary Table S2). Also, a higher proportion of patients who survived at 180 days after ICU admission received emergency hemodialysis, ECMO, CABG, emergency surgery, and dopamine injection, whereas a lower proportion of surviving patients used ventilators (Supplementary Table S2). When considering the potential influence of covariates, the occurrence of AKI seemed to be negatively correlated with 180-day mortality (aOR 0.67, 95% CI 0.51–0.88, *p* = 0.003; aOR 0.60, 95% CI 0.39–0.92, *p* = 0.019; aOR 0.55, 95% CI 0.37–0.83, *p* = 0.004, for AKI stage 1–3, respectively) whereas AKD, especially stage 3, was a detrimental factor for 180-day mortality (aOR 1.96, 95% CI 1.25–3.06, *p* = 0.003) (Table 4). Compared to patients with AKI without AKD, patients who had no AKI but suffered from AKD after ICU admission had the highest risk of 180-day mortality (aOR 2.25, 95% CI 1.71–2.97; *p* < 0.001), even higher than that in patients with AKI and AKD (aOR 1.34, 95% CI 1.00–1.78; *p* = 0.047). The discrimination ability of the regression model for the prediction of 180-day mortality was within an acceptable range (area under curve = 0.702; 95% CI 0.667–0.737) (Figure 3).

**Table 4:** Univariable and multivariate logistic regression of different combinations of AKD and AKI associated with 180-day mortality.

	Univariable			Multivariable		
Covariates	OR	95% CI	*p* value	aOR	95% CI	*p* value
AKD stage						
Stage 0	ref			ref		
Stage 1	1.14	(0.77–1.69)	0.506	0.95	(0.64–1.42)	0.806
Stage 2	1.56	(1.02–2.36)	0.038	1.41	(0.92–2.16)	0.117
Stage 3	2.10	(1.37–3.21)	0.001	1.96	(1.25–3.06)	0.003
AKI stage						
Stage 0	ref			ref		
Stage 1	0.41	(0.33–0.51)	<0.001	0.67	(0.51–0.88)	0.003
Stage 2	0.41	(0.28–0.61)	<0.001	0.60	(0.39–0.92)	0.019
Stage 3	0.39	(0.28–0.56)	<0.001	0.55	(0.37–0.83)	0.004
Scenarios						
AKI without AKD	ref			ref		
AKI with AKD	1.47	(1.11–1.95)	0.007	1.34	(1.00–1.78)	0.047
No AKI with AKD	2.84	(2.27–3.57)	<0.001	2.25	(1.71–2.97)	<0.001

AKD, acute kidney disease; AKI, acute kidney injury. Adjusted OR (aOR) was the risk of 180-day mortality of different combinations of AKI/AKD when considering gender, age group, duration of hospitalization, comorbidities, qSOFA, albumin, total bilirubin, lactate, prothrombin, procedures and medications performed during hospitalization.

## 4. Discussion

To study the clinical characteristics and outcomes of AKD, we analyzed 11,045 AKI survivors, and 5,178 AKD patients without prior AKI who were admitted to ICU. Several critical risk factors for the occurrence of AKD among AKI patients were identified. The main findings were as follows: first, the incidence of AKD after AKI was 22.5% (3,797 of 16,855). The overall 180-day mortality rate was 2.3% (88 of 3,797) in patients with AKD after AKI, and 4.4% in patients with AKD without AKI (227 of 5,178), which was consistent with previous studies (2, 12, 21–24). Second, several independent risk factors for the development of AKD were identified: severity of AKI, female gender, being older, preexisting early CKD, underlying chronic liver disease, malignancy, and the use of emergency hemodialysis, intra-aortic balloon pumps, dopamine, and longer hospitalization stays. In contrast, male gender, underlying hypertension, cardiovascular disease, higher qSOFA scores, higher lactate levels, the use of ECMO and ventilators, receiving emergency surgery, and admission to surgical ICU were negatively correlated with the development of AKD. Third, when compared to AKI without AKD, the occurrence of AKD after AKI, but not AKD without prior AKI, was associated with a significantly increased risk of 180-day mortality in critically ill patients.

Renal dysfunction impacts innate and adaptive immunity, autoregulation, and vasodilation response, and reduces tolerance to the side effects of drugs (4, 25, 26). Previous studies have proposed that pre-existing CKD can modify the risks of clinical outcomes subsequent to AKI (11, 27). Patients with preexisting CKD were more susceptible to various acute insults, possibly leading to oliguria, fluid overload, electrolyte, or acid–base disturbances, and uremia. Such patients might fulfill the criteria for dialysis or severe AKI when exposed to less severe damage (4). Our study demonstrated that preexisting CKD, the severity of AKI, and the use of emergency hemodialysis were independent risk factors for the development of AKD. On the other hand, the incidence of AKD in patients admitted to the surgical ICU (25.0%, 178 of 712) was much lower than in patients admitted to non-surgical ICU (35.0%, 3,619 of 10,333; *p* < 0.001). The patients who received emergency surgery (31.6%, 1,657 of 5,242) also had significantly lower incidence of AKD than the total patient cohort (34.4%, 3,797 of 11,045; *p* < 0.001). Our data were in line with evidence from previous studies showing that surgical etiology was an independent predictor of better outcomes, such as high survival and renal recovery (14, 28). Interestingly, patients with cardiovascular disease, high qSOFA scores, higher lactate levels, and who used ECMO and ventilators, also had significantly lower incidence of AKD in the current study. In the literature, the occurrence of AKD has been connected to the combined effects of structural kidney damage and less responsive renal repair (2). Considering that these are well-known risk factors for extremely grave outcomes, the paradoxically protective effect of these factors on the development of AKD might be attributed to the competing risk of death. This interpretation is consistent with previous reports (14, 22, 29–32).

AKD is considered an important transition period in the AKI-to-CKD continuum (33). The severity of AKD has been reported to be significantly correlated to increased risk of adverse outcomes (14, 15, 22, 34–36). However, several previous studies have reported conflicting results (22, 33, 37–39). Impressively, our study showed that most AKI survivors (55.5%, 7,248 of 13,069) experienced complete renal recovery and did not develop AKD within 7–90 days after the AKI episode. The occurrence of AKD added limited prognostic information for 180-day mortality after AKI (aOR 1.33, 95% CI 1.00–1.77, compared to AKI without AKD). Nevertheless, we found that critically ill patients who had no AKI but later developed AKD (aOR 2.25, 95% CI 1.71–2.97) had a greater risk than the patients who developed AKD after AKI (aOR 1.34, 95% CI 1.00–1.78; *p* = 0.047). These findings indicate that the presence of AKD may provide limited information for risk stratification of survivors among critically ill patients with AKI but could predict prognosis in survivors without AKI.

Based on these findings, we recommend continued monitoring of renal function even in critically ill patients who do not meet the diagnostic criteria for AKI after an acute renal insult. More attention should also be given to those who develop AKD without AKI (i.e., sCr levels <1.5 times baseline within 7 days of an episode that increased to >1.5 times baseline 7–90 days after the episode). The occurrence of AKD without AKI might signify disease progression of subclinical AKI, which is not detected by current sCr-based AKI criteria. Clinical physicians should incorporate the comprehensive kidney health assessments set out by the ADQI 22 working group into clinical practice for critically ill patients who do not meet criteria for AKI after an acute insult but are at risk of AKD (40). Targeted management using an intensive care program, including the avoidance of exposure to nephrotoxic agents and unnecessary drugs, and regular monitoring of renal function, might help reduce the incidence of AKD and the risk of subsequent adverse outcomes in critically ill patients surviving 7 days after an acute insult (41).

While our study yielded encouraging results, it is important to acknowledge its potential limitations. First, the patient population was heterogeneous, with varying causes of acute kidney injury (AKI), the severity of kidney damage, and accompanying chronic comorbidities. For this reason, we conducted a multivariable analysis to better understand these variables’ clinical impact. Second, due to the retrospective nature of our study, determining the exact onset time of each AKI episode was challenging. To address this, we defined the date of AKI onset as the day within the first seven days of ICU admission when the lowest serum creatinine (sCr) measurement was recorded. While this approach may not accurately capture the ongoing progression of AKI or incomplete renal recovery after AKI, recent research from the same database suggests that our approach likely had a low risk of bias (42). Nevertheless, subclinical AKI may be missed due to a lack of biomarkers other than sCr in the database (43–45). Third, community-acquired AKI may have been misdiagnosed as chronic kidney disease (CKD) due to our use of sCr data collected during admission, which could have led to an underestimation of the true incidence of AKI. Forth, information about blood transfusions was not available in our database. While the demand for blood transfusions may be low in this population, the lack of information on this variable may have influenced our outcomes. Finally, it is essential to note that logistic regression models have inherent limitations in their predictive accuracy, and the lack of external validation from an independent cohort is a significant concern. Also, the study only involved patients of a single ethnicity, which limits the generalizability of the findings to other hospitals with different patient populations. Future studies should aim to validate our findings in a separate cohort of patients to increase the reliability and generalizability of our results.

## 5. Conclusion

In the present study, approximately a quarter of critically ill AKI patients developed AKD, and another quarter died within 180 days. Our analyses showed that AKI stage 2–3, underlying early CKD, chronic liver disease, malignancy, and use of emergency hemodialysis were independently associated with increased risk of AKD development. Although the occurrence of AKD did not add prominent prognostic information for risk stratification of survivors among critically ill patients with AKI, it could predict prognosis in survivors without AKI.

## Data availability statement

The raw data supporting the conclusions of this article will be made available by the authors, without undue reservation.

## Ethics statement

The studies involving human participants were reviewed and approved by the Institutional Review Board of the Chang Gung Medical Foundation. Written informed consent for participation was not required for this study in accordance with the national legislation and the institutional requirements.

## Author contributions

H-CP and H-YC contributed to data conception, design, and interpretation. H-YC, H-MC, Y-TH, and H-CP contributed to collecting data and manuscript drafting. Y-CC, J-TF, and H-CP provided patient information, participated in the design and coordination, and helped draft the manuscript. H-YC, H-CP, and Y-CC provided intellectual content of the work and were involved in editing and revising the manuscript. All authors discussed, contributed to, and approved the final manuscript version.

## Funding

We thank financial support from the Ministry of Science and Technology, Taiwan (MOST 107-2314-B-182A-019-MY3), and in part from the Chang Gung Medical Foundation (CMRPG1M0041, CMRPG1M0121, and CORPG1L0041).

## Conflict of interest

The authors declare that the research was conducted in the absence of any commercial or financial relationships that could be construed as a potential conflict of interest.

## Publisher’s note

All claims expressed in this article are solely those of the authors and do not necessarily represent those of their affiliated organizations, or those of the publisher, the editors and the reviewers. Any product that may be evaluated in this article, or claim that may be made by its manufacturer, is not guaranteed or endorsed by the publisher.
