# Corrigendum: Risk factors and 180-day mortality of acute kidney disease in critically ill patients: a multi-institutional study

**DOI:** 10.3389/fmed.2023.1220006

**Published:** 2023-05-23

**Authors:** Heng-Chih Pan, Hsing-Yu Chen, Hui-Ming Chen, Yu-Tung Huang, Ji-Tseng Fang, Yung-Chang Chen

**Affiliations:** ^1^Chang Gung University College of Medicine, Taoyuan, Taiwan; ^2^Division of Nephrology, Department of Internal Medicine, Keelung Chang Gung Memorial Hospital, Keelung, Taiwan; ^3^Graduate Institute of Clinical Medical Sciences, College of Medicine, Chang Gung University, Taoyuan, Taiwan; ^4^Division of Chinese Internal Medicine, Center for Traditional Chinese Medicine, Chang Gung Memorial Hospital, Taoyuan, Taiwan; ^5^School of Traditional Chinese Medicine, College of Medicine, Chang Gung University, Taoyuan, Taiwan; ^6^Center for Big Data Analytics and Statistics, Chang Gung Memorial Hospital, Linkou Medical Center, Taoyuan, Taiwan; ^7^Division of Nephrology, Department of Internal Medicine, Linkou Chang Gung Memorial Hospital, Taoyuan, Taiwan

**Keywords:** acute kidney injury, acute kidney disease, chronic kidney disease, risk factor, survival

In the published article, an author name was incorrectly written as Heng-Chi Pan. The correct spelling is Heng-Chih Pan.

The authors apologize for this error and state that this does not change the scientific conclusions of the article in any way. The original article has been updated.

