# Supplementary material for: Risk factors and 180-day mortality of acute kidney disease in critically ill patients: A multi-institutional study
Source: Front Med (Lausanne). 2023 Apr 17;10:1153670. doi: 10.3389/fmed.2023.1153670 (PMC10149804; doi:10.3389/fmed.2023.1153670)
Supplement: Supplementary file 1 [file Table_1.DOCX]

**Supplementary Table S1.** Diagnostic codes used in this study.

| Disease | ICD-9-CM codes | ICD-10 codes |
| --- | --- | --- |
| Diabetes mellitus | 250 | E10, E11 |
| Hypertension | 401, 402 | I10, I11 |
| Cardiovascular disease | 410, 427.31, 428, 430, 431, 433, 434 | I21, I48.0, I48.2, I48.91, I50, I60, I61, I65, I66 |
| Chronic kidney disease | 580, 581, 582, 583, 584, 585, 586, 587, 588, 589 | I12, I13, N00–N05, N07, N11, N14, N17, N18, N19, Q61 |
| Chronic liver disease | 571 | K70 |
| Malignancies | 140–175, 179–208 | C00–C97, D00–D48 |

| **Supplementary Table S2.** Differences in demographic characteristics between cases that survived and those who died | | | | | |
| --- | --- | --- | --- | --- | --- |
| Covariates | Survived  (n = 15,793) | | Died  (n = 430) | | p-value |
| AKI/AKD combinations |  |  |  |  | < 0.001 |
| AKI without AKD | 7,133 | 45.2% | 115 | 26.7% |  |
| AKI with AKD | 3,709 | 23.5% | 88 | 20.5% |  |
| AKD without AKI | 4,951 | 31.3% | 227 | 52.8% |  |
| Gender |  |  |  |  | 0.570 |
| Female | 5,593 | 35.4% | 158 | 36.7% |  |
| Male | 10,200 | 64.6% | 272 | 63.3% |  |
| Age group |  |  |  |  | 0.162 |
| < 70 years | 9,309 | 58.9% | 239 | 55.6% |  |
| ≥ 70 years | 6,484 | 41.1% | 191 | 44.4% |  |
| Hospitalization |  |  |  |  | < 0.001 |
| < 25 | 7,242 | 45.9% | 158 | 36.7% |  |
| ≥ 25 | 8,551 | 54.1% | 272 | 63.3% |  |
| Cause |  |  |  |  | 0.001 |
| Non-surgical ICU | 14,724 | 93.2% | 418 | 97.2% |  |
| Surgical ICU | 1,069 | 6.8% | 12 | 2.8% |  |
| Comorbidities |  |  |  |  |  |
| DM | 5,594 | 35.4% | 153 | 35.6% | 0.945 |
| Hypertension | 7,538 | 47.7% | 207 | 48.1% | 0.867 |
| Cardiovascular diseases | 5,649 | 35.8% | 124 | 28.8% | 0.003 |
| Chronic liver diseases | 2,485 | 15.7% | 93 | 21.6% | 0.001 |
| Malignancies | 2,422 | 15.3% | 136 | 31.6% | < 0.001 |
| qSOFA |  |  |  |  | 0.519 |
| Low Risk (0–1) (n, %) | 13,791 | 87.3% | 380 | 88.4% |  |
| High Risk (2–3) (n, %) | 2,002 | 12.7% | 50 | 11.6% |  |
| Procedures |  |  |  |  |  |
| Emergency hemodialysis (n, %) | 397 | 2.5% | 9 | 2.1% | < 0.001 |
| ECMO (n, %) | 846 | 5.4% | 6 | 1.4% | 0.033 |
| Ventilator use (n, %) | 6,539 | 41.4% | 234 | 54.4% | < 0.001 |
| CABG (n, %) | 1,271 | 8.0% | 6 | 1.4% | 0.002 |
| IABP (n, %) | 648 | 4.1% | 4 | 0.9% | 0.060 |
| Emergency surgery (n, %) | 6,668 | 42.2% | 137 | 31.9% | <0.001 |
| Medication |  |  |  |  |  |
| Norepinephrine (n, %) | 1,463 | 9.3% | 48 | 11.2% | 0.181 |
| Dopamine (n, %) | 2,691 | 17.0% | 42 | 9.8% | < 0.001 |

Abbreviations: AKD, acute kidney disease; CABG, coronary artery bypass graft; CCU, coronary care unit; ECMO, extracorporeal membrane oxygenation; IABP, intra-aortic balloon pump; MICU, medical intensive care unit; qSOFA, Quick Sequential Organ Failure Assessment; SD, standard deviation; SICU, surgical intensive care unit
